# Vertical equity of healthcare in Taiwan: health services were distributed according to need

**DOI:** 10.1186/1475-9276-12-12

**Published:** 2013-01-31

**Authors:** Shiow-Ing Wang, Chih-Liang Yaung

**Affiliations:** 1 National Environmental Health Research Center, National Health Research Institutes, Miaoli County, Taiwan; 2 Department of Healthcare Administration, College of Health Science, Asia University, Taichung, Taiwan

## Abstract

**Introduction:**

To test the hypothesis that the distribution of healthcare services is according to health need can be achieved under a rather open access system.

**Methods:**

The 2001 National Health Interview Survey of Taiwan and National Health Insurance claims data were linked in the study. Health need was defined by self-perceived health status. We used Concentration index to measure need-related inequality in healthcare utilization and expenditure.

**Results:**

People with greater health need received more healthcare services, indicating a pro-need character of healthcare distribution, conforming to the meaning of vertical equity. For outpatient service, subjects with the highest health need had higher proportion of ever use in a year than those who had the least health need and consumed more outpatient visits and expenditures per person per year. Similar patterns were observed for emergency services and hospitalization. The concentration indices of utilization for outpatient, emergency services, and hospitalization suggest that the distribution of utilization was related to health need, whereas the preventive service was less related to need.

**Conclusions:**

The universal coverage plus healthcare networking system makes it possible for healthcare to be utilized according to need. Taiwan’s experience can serve as a reference for health reform.

## Introduction

Health equity is an overarching goal for health care reform around the world. Healthcare is the most commonly cited example of a commodity that ought to be distributed according to need [1]. Numerous countries have endorsed a policy objective that access and use of healthcare should be based on need, not the ability to pay [2]. However, health needs are greatly unmet in vulnerable groups in most societies. The Institute of Medicine (IOM) estimates that at least 18,000 Americans die prematurely each year because they lack health insurance to get appropriate healthcare [3]. Poverty is associated with increased chronic diseases, not seeking medical care and premature death [4-6]. The reasons women do not seek care in the case of obstetric emergencies including lack of knowledge, financial costs, attitudes of family members and religion [7]. Many studies reveal that patient use of services tends to decline with distance [8,9], or transport costs [10,11]. Evidence is accumulating that financial, geographic, or cultural barriers contribute greatly to the phenomenon of health unmet [7]. We are still far from “health for all”.

Sachs [12] emphasized that structural change in health services are needed to overcome the substantial barriers to access for vulnerable groups. Researchers point out that vertical equity is as one way of proceeding [13-16].Equity in health has been conceptualized and defined in several ways. Two main forms of health equity are identified, vertical equity (people with greater health needs should receive more healthcare than those with lesser needs), and horizontal equity (equal treatment for equivalent needs). Most studies in the assessment of healthcare utilization have mainly focused on horizontal equity [17-22] and as a consequence have tended to overlook vertical equity. However, as more and more discussion and inspiration, the meaning of health equity has expanded to creating equal opportunities for health. Allocation resource base on need become accepted way to assure that sicker people (imply he has higher need to restore the opportunity) will receive more health services regardless of extraneous circumstances such as race or level of income. Studies relevant to the vertical equity of healthcare utilization are scarce [13-15], one major difficulty arises because of the fact that it is hardly to find a country with minimal barriers for its citizen to get health services to conduct the research. Vertical equity of healthcare utilization cannot be easily tested under conditions that barriers for healthcare use exist. Hence, the nearly barrier-free healthcare system in Taiwan became an ideal place to test the theory of vertical equity.

To achieve the goal of health for all and to eliminate financial barriers to health care services, the government of Taiwan launched the National Health Insurance (NHI) program to provide universal medical care coverage on March 1, 1995. Since then, the coverage rate has climbed steadily and reached 99% by the end of 2009 [23]. The NHI program is a mandatory, single-payer social health insurance system. The program offers comprehensive benefits, including inpatient and ambulatory care, dental services, traditional Chinese medicine, physical rehabilitation, home nursing care, and preventive services to every insured. Citizens insured under the NHI program have the freedom of choosing any contracted facilities/institutions for his/her care need. No “gatekeeper” controls the utilization and no waiting list exists. The revenue for NHI relies on payroll-based premiums, government funding and out-of-pocket payments for services. A copayment fee ranging from US$ 2~12 normally charged for each clinical visit depending on the level of the facility. Those who cannot afford their premiums are eligible for assistance (including premium subsidies, relief fund loans, and sponsorship referrals) from the Bureau of National Health Insurance (BNHI). Copayments are also waived for the poor, veterans, and aborigines to ensure that health expenditures do not discourage patients from seeking necessary medical need [23]. There is pro-poor inequality in the probability of visiting a doctor, but the distribution of total medical expenditure is progressive with income in Taiwan [7,20].

Meanwhile, the geographic barriers to healthcare are relatively low. In order to balance medical resources, the Department of Health (DOH) also launched the Establishment of Medical Care Network in Taiwan in July 1985. The project divided the Taiwan area into 17 medical care regions, each of which served as the basic unit for developing medical manpower, facilities, and an emergency care network. These 17 medical regions were further subdivided into 63 sub-regions based on population density, geographic location, and transportation facilities. Each sub-region was equipped with regional or district hospitals, as well as primary medical care units. The Medical Care Network project also sought to distribute medical resources more evenly by restricting the establishment or expansion of hospitals in regions with plentiful medical resources and by setting up a Medical Care Development Fund to encourage the private sector to establish health care institutions in regions lacking sufficient medical facilities [24]. Currently, the program focuses on setting up integrated medical care service and quality improvements. The BNHI also introduced an integrated delivery system (IDS) to improve services in remote mountainous areas and outlying islands.

As a result of these efforts, we can assume that Taiwan’s health system have an environment with minimal barriers to healthcare and is an ideal condition for testing the vertical equity hypothesis “people with greater health needs should receive more healthcare than those with lesser needs”. Thus, the purpose of the present study was to test the hypothesis that under Taiwan’s universal access health system, healthcare utilization is determined by health need.

## Methods

### Sources of data

Data used for this analysis were from the 2001 National Health Interview Survey (NHIS) of Taiwan that was conducted by the National Health Research Institute and the Bureau of Health Promotion, Taiwan. The NHIS was a government-sponsored project for gathering information on health status, medical utilization, health behavior, and quality of life of the Taiwanese population. It employed a multistage sampling design to draw a population-representative sample. In all, 6,592 households (26,658 persons) were selected. Each member of the selected household participated in a face-to-face interview by trained interviewers. Ninety-one percent of the selected households and 94.2% of the individuals completed the survey. Further detail of the design, methods, and procedure is available [25].

To enhance academic study, the NHRI has linked data of the participants with their healthcare records (2001) maintained by the BNHI. The original NHIS survey and the linking to the NHI data from which information used in the current study was obtained had ethical approval from the review board composed of government-appointed representatives. All data were free of personal identification and personal privacy was strictly maintained according to the guidelines enacted in the Declaration of Helsinki.

### Study subjects

Age is an important factor in determining health care needs. Health needs of adolescents and the elderly are significantly different. Therefore to avoid the interference caused by the age, the study only included people aged 40–64.

### Study variables

#### Health need

According to Donabedian [26], health needs begin with a state of health expressed in the need for services conceived in response to such states. Previous investigations have found that self-perceived health can predict mortality, morbidity, and healthcare utilization [27-34] independently of objective indicators. Wolinsky and Johnson [35] suggested that the ability of self-perceived health to predict health status may be due to individuals’ ability to detect their own medical conditions in the preclinical stage when the conditions cannot be medically diagnosed. Self-perceived health status reflects a person’s integrated perception of health, including its biological, psychological and social dimensions [36], which are inaccessible to the observations of doctors. Thus, self-perceived health status can be considered as one of the best indicator of need for health care resources [37-40].

In the present study self-rated health condition was assumed to reflect health need. Health need was derived from the response to the question “In general, what do you think about your current health status?” with a five-level Likert item (excellent, good, fair, somewhat poor and poor) in the NHIS questionnaire. Those who self-rated as having “excellent” health status were assumed to have lowest health need, whereas those who self-rated as having “poor” health were assumed to have highest health need.

#### Healthcare utilization

The main outcome measure was healthcare utilization including the annual number of physician visits and the medical expenditures. In addition, the determinants of each type of healthcare (outpatient, emergency, preventive) vary considerably. We further divided healthcare utilization into outpatient, emergency, preventive services and hospitalization to figure out whether the result of test “healthcare utilization is determined by health need” will be different in different type of care.

### Statistical analyses

First, we described the characteristics of subjects according to health need. Then, we calculated health care utilization stratified by health need to determine the differences in health care utilization. The concentration curve and related concentration index (CI), an analysis which is widely used to assess the degree of inequality in the distribution of a health variable [41], was used to measure need-related inequality in health care utilization according to the formula proposed by Fuller and Lury [42]:

(1) $$\begin{array}{l} \mathrm{CI}=\left(p_{1}L_{2}{-p}_{2}L_{1}\right)+\left(p_{2}L_{3}{-p}_{3}L_{2}\right)+\ldots \\ \;+\left(p_{T-1}L_{T}{-p}_{T}L_{T-1}\right) \end{array}$$

where *p* is the cumulative percentage of the sample ranked by health need status, *L* is the corresponding concentration curve ordinate, and *T* is the number of health need groups. *CI* lies in the range from −1 to 1, with a negative value indicating a pro-need character of healthcare distribution. The calculation of *CI* and its standard error was based on Excel resources provided by the World Bank [43].

A concentration curve plots the cumulative proportion of the individuals under consideration ranked by health need, beginning with the very-high-need (poorest health) and ending with the very-low-need (x-axis), against the cumulative proportion of the health care utilization (y-axis) being measured. Usually, “a 45-degree diagonal line” is considered the line of equality. However, this study was to test the hypothesis of “unequal treatment for unequal need” meaning that persons with poorer health status would have greater health need and greater care utilization. Therefore, a curve lies above the line of equality would be expected to have a negative concentration index, indicating that health care utilization is higher for those having higher health need (pro-need).

## Results

Table 1 shows the basic characteristics of subjects and the distribution of self-perceived health status according to these characteristics. The sample included slightly more women (53.4%); 53.2% were 40–49 years old, 33.9% were 50–59 years old, and the rest were 60–64 years old; 60% had less than 9 years of formal education, and approximately a quarter reported no monthly income. Among 4,880 subjects, 2.9% rated themselves “excellent health”, 24.5% rated “good health”, 26.4% rated “fair health”, 38.3% rated “somewhat poor health” and 7.9% rated “poor health”. The perceived health status was significantly different between genders, and among various age, education, or income levels (p<0.001). Greater proportions of male or younger subjects (40–49 years old) perceived themselves having good health. Greater proportions of subjects with more years of education or higher income also rated themselves better health.

**Table 1:** Distribution (%) of subjects according to health need^1^ stratified by gender, age, level of education and monthly income (N=4880)

**Distribution of subjects by health need (%)**						
**Variables**	**Total n**	**Very low**	**Low**	**Moderate**	**High**	**Very high**
		**(n=141)**	**(n=1195)**	**(n=1290)**	**(n=1867)**	**(n=387)**
Gender^***^						
Male	2276	4.0	28.5	26.8	33.9	6.7
Female	2604	1.9	21.0	26.1	42.1	9.0
Age (y)^***^						
40-49	2596	3.5	28.9	28.9	33.0	5.7
50-59	1654	2.1	20.5	25.4	43.0	9.0
60-64	630	2.4	16.8	19.0	47.5	14.3
Formal education (y)^***^						
≤6	2078	2.0	18.4	21.8	46.7	11.1
7-9	795	3.9	26.0	27.7	36.5	5.9
10-12	1097	3.5	28.9	30.4	31.9	5.3
>12	821	3.4	33.7	32.8	25.7	4.4
Monthly income (US$)^***^						
0	1237	2.4	18.9	22.9	42.2	13.6
1-300	517	1.9	11.8	21.7	51.6	13.0
301-600	770	2.7	20.5	23.8	46.1	6.9
601-1200	1163	2.6	27.6	29.8	34.7	5.2
1201-1800	646	4.3	34.1	31.0	28.3	2.3
>1800	507	3.7	38.8	31.1	22.5	3.9

^1^The level of health need is based on self-rated health status. Those who self-rated as having excellent status are assumed to have very low health need whereas those who self-rated as having poor status are assumed to have very high need.

***p<0.001 based on Chi-square test.

Table 2 shows healthcare utilization and expenditure classified according to various levels of health need. The utilization and expenditure were significantly different between need levels (p<0.001) in outpatient, emergency, hospitalization and preventive services. For outpatient services, subjects who had the highest health need had higher proportion of ever use in a year compared to those who had the least health need (98.2% vs. 92.2%) and consumed more outpatient visits (28.3 vs. 10.4) and expenditures ($1,112 vs. $191) per person per year. Similar patterns were observed in emergency services and hospitalization.

**Table 2:** Average utilization (mean±SD) and expenditure (mean±SD) of various types of healthcare services according to levels of health need (N=4880)

**Level of need**	**Outpatient care**			**Emergency care**			**Hospitalization**			**Preventive care**		
	**% used^1^**	**# of visit^2^**	**Expenditure^3^**	**% used^1^**	**# of visit^2^**	**Expenditure^3^**	**% used^1^**	**# of visit^2^**	**Expenditure^3^**	**% used^1^**	**# of visit^2^**	**Expenditure^3^**
Very low (n=141)	92.2	10.4±10.7	191±241	10.6	0.13±0.41	5±22	0.01	0.02±0.19	60±600	11.3	0.2±0.6	2±6
Low (n=1195)	93.1	12.5±12.1	280±472	11.1	0.16±0.58	8±33	0.05	0.08±0.57	98±733	17.9	0.3±0.7	3±6
Moderate (n=1290)	95.3	14.9±12.3	318±368	13.5	0.19±0.62	11±49	0.08	0.10±0.43	131±1057	23.3	0.4±0.9	4±7
High (n=1867)	97.8	19.1±15.7	541±1472	14.8	0.21±0.66	13±56	0.09	0.13±0.53	174±1462	26.8	0.5±0.9	4±8
Very high (n=387)	98.2	28.3±23.8	1112±2432	19.4	0.38±1.12	35±140	0.19	0.41±1.45	620±3637	28.2	0.5±0.8	4±8
Pearson χ^2^/ F	50.8^***^	107.23^***^	45.26^***^	20.25^***^	8.58^***^	16.43^***^	83.16^***^	21.44^***^	9.543^***^	48.76^***^	11.41^***^	13.10^***^

^1^Percent of patients in group used the service.

^2^Average number of visit (mean±SD)/person.

^3^Average expenditure in US$ (including co-payment and other expenses related to the services) (mean±SD)/person.

***p<0.001.

**Figure 1:**
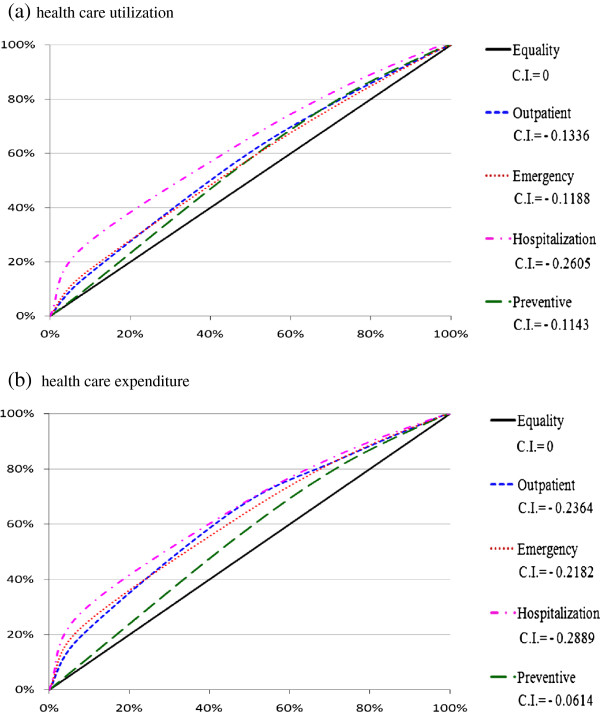
**Concentration curve and Concentration Index (C.I.) of healthcare utilization and expenditure for health need.** Note: x-axis is cumulative proportion of population ranked by health need, beginning with the very-high-need (poorest health). y-axis is cumulative proportion of health utilization or expenditure.

The concentration curves of healthcare utilization and healthcare expenditure both lay above the line of equality and the concentration indices are negative, indicating a pro-need character of healthcare distribution (Figure 1). For utilization, the C.I. were –0.1336 for outpatient, –0.1188 for emergency services, –0.2605 for hospitalization and –0.1143 for preventive services, respectively. For expenditure, the C.I. were –0.2364 for outpatient, –0.2182 for emergency services, –0.2889 for hospitalization and –0.0614 for preventive services, respectively.

## Discussion

### Healthcare utilization determined by health need is well revealed

We used a rather open access system to examine the relationship between health need and healthcare utilization. Results of the present study show that the distribution of healthcare utilization and expenditure is pro-need character in Taiwan. People who have greater health need use more healthcare services, conforming to the principle of vertical equity.

Healthcare utilization determined by health need is well revealed in certain area of health services. A clear gradient of utilization and expenditure were found according to the levels of health need in outpatient, emergency services and hospitalization, especially in hospitalization. In Taiwan, we don’t have a family physician to play a gatekeeper role, nor does it limit the number of visit per year. The system appears to have an open access to outpatient or emergency services. By contrast, the need for hospitalization is judged by the professionals rather than the patient. The findings of present study showed the consistence of self-perceived health and physician’s assessment, inferred that self-rated health is valid as a single measure of overall health.

Compares to the hospitalization services, the difference in preventive services utilization and expenditure between lowest health need and highest need group is smaller. The concentration index is close to zero, meaning that there is no need-related inequality in preventive services. The results were similar with other studies [44,45]. The preventive services aim to improve health for all, while other health services aim to “treat” patients. They have different purposes and target groups. The sustainability of preventive services empower people be more self-determining [46]. Equitable access to preventive health services is widely promoted as one of the most feasible remedies to reduce health disparities [47-50]. Thus, we believe that the need for preventive services should nearly the same regardless of health status.

### Taiwan’s experience

In a virtually barrier-free system like Taiwan, vertical equity for those aged 40–64 does exist. We believe this is mainly related to four factors: universal coverage, comprehensive benefit, low copayment, and networking health system. Although health services can only contribute 10% of health [51], universal access to health services is fundamental to assure people that health needs can be met equitable. Various studies in Taiwan suggest that universal access health system greatly removed the financial barrier to care [52], narrow down health disparity in life expectancy [53], reduce the difference of health status or utilization between remote and non-remote populations [10,11,54], and improve resource allocation equally [55,56]. Low income groups earn higher benefit from the universal access health system and bear relatively low premium burden [24]. Despite Buchanan proposed the concept of “decent minimum of health care” [57], it remains no consensus about what we should provide. In Taiwan, the benefits of NHI are comprehensive which allow a variety of health need can be met. In addition, low copayment avoids patients to delay medical treatment due to financial consideration, and provides equal opportunities for disadvantaged groups to receive quality healthcare. The networking system reduced the gap between urban and rural areas, thereby significantly reducing the medical geographical obstacles. Accordingly, the contribution of an open access health system to increasing vertical equity in healthcare utilization is conclusive.

No system is perfect. One of the criticized situation in Taiwan is that doctors spending too little time in patients. When a doctor can only spend 2–3 minutes for an outpatient visit, it cannot help but worry about the quality of care. It is difficult to catch patient’s needs completely in such a short period of time. Fortunately, Taiwan enacted a number of clinical guideline, so that physicians can follow the guideline to provide services. The regulations for NHI reimbursement may also standard doctor’s diagnosing and treating behavior. People in Taiwan have more outpatient visits per year compared to their counterparts in Western nations. Even for persons who perceive themselves having excellent health, their annual outpatient visits (10.3) is higher than that in the United States, Canada or the United Kingdom [16]. The accessibility of Taiwan’s health care system is the main reason contributing to this phenomenon. The statement that “Healthcare utilization can exceed basic needs” [58] is fully displayed in Taiwan. However, with resources constraint, no country can afford unguarded use of health resources. Health policy planners are facing the dilemmas of satisfying needs and scarcity of resources.

### Strength of the study

Scholars recommend that “characteristics of the health care system that influence health equity” is one of highest priority should be given to research [59] which is ongoing in the present study.The concept of “vertical equity” is still in the stage of theoretical discussion. We provided an empirical test of the principle of unequal treatment for unequal need can be achieved under a rather open access system. We also used the familiar method which is widely used to assess horizontal equality to clarify the vertical equity concept. Results from this study can serve as a basis for further research to evaluate the health equity, clarify the causes of the gap between need groups, and to stimulate additional research to further investigate vertical equity. Results will also be useful to health planners in designing policies or programs for eliminating disparities and achieving the goal of health equity.

### Study limitations

Several limitations must be mentioned. First, although self-rated health can be considered as an indicator of the intensity of health need, people perceive their status differently. Previous studies have shown that self-rated health was influenced by individual demographic factors (such as age, gender, education or income), personal health practices or social psychological resources (including social support, self esteem) [60]. In addition, health need is unique and distinctly vary from one person to another. The value of health or expectation differs among people. Therefore, it would be useful to confirm the consistency of results with other measurements, such as physical examination which illness is based on medical diagnose, or an analysis of diagnostic categories presented by patients in hospitals [61]. Furthermore, Likert items are ordinal scale. It is not indicate that the difference between “excellent” and “good” is the same size as the difference between “somewhat poor” and “poor”. We treat it as interval scale while calculating healthcare utilization, which may have biased conclusion.

Second, this study used a broader perspective to explore the relationship between health needs and healthcare utilization. This approach makes different diseases mixed together which may interfere our understanding of the truth. We recommend further researches can focus on a single disease. In addition, this is a cross sectional study with only a one-year observation. One might argue that it is a forced interpretation to attribute such performance to the universal coverage system. However, according to several studies [10,11,52], we believe that universal coverage health system did have its contribution on the distribution of healthcare services related to health need.

Although the NHIS is representative of the population of Taiwan, only 88% of subjects in this study agreed to have their data linked to NHI database, which may produce bias. Furthermore, for demonstration purposes, the present study selected subjects aged 40–64. These subjects occupied for only one-third of all surveyed ones which may not represent the whole system in Taiwan. We will conduct follow-up studies with other age groups to confirm the consistency of results. From 2001 to 2010 the elderly population in Taiwan increased more than 26% [62], more advanced technologies were introduced in the medical service, the NHI covered more services in the reimbursement system [23]. Those changes might have altered the distribution of health resources. Further researches can integrate more materials to explore the trend of distribution of healthcare services according to health need.

Despite national health insurance makes healthcare accessible to everyone, personal preferences should be taken into account. What is considered a need can be importantly determined by cultural beliefs, attitudes, conventions, and preference [63]. Some people may choose to receive folk remedies or natural cures, services not covered by NHI. Personal health habits, attitudes, living environment and life-style may also influence the development of disease. This study was unable to integrate these non-medical factors into analysis.

## Conclusions

The present study demonstrate that people with greater health need received more healthcare services, the concentration curve lies above the line of equality, indicating that healthcare utilization is higher for those having higher health need, confirmed the meaning of vertical equity under a rather open access health system. Health is one of the fundamental human rights, and government should be accountable for reaching the goal of “health for all”. Under the resources constrain, allocate health resources according to need would be an effective and moral way. Taiwan’s experience can serve as a reference for health reform.

## Competing interests

The authors declare that they have no competing interests.

## Authors’ contributions

SIW and CLY participated in the design of the study. SIW performed the statistical analyses, and CLY supervised in acquisition of data. SIW and CLY jointly drafted the present manuscript version. All authors read and approved the final manuscript.
